# A Treatment Strategy for Severe Legionella Pneumonia Using Veno-Venous Extracorporeal Membrane Oxygenation

**DOI:** 10.7759/cureus.75528

**Published:** 2024-12-11

**Authors:** Kosuke Otake, Takashi Tagami, Junichi Inoue, Kiyoshi Matsuda, Shoji Yokobori

**Affiliations:** 1 Department of Emergency and Critical Care Medicine, Nippon Medical School Musashi-Kosugi Hospital, Kawasaki, JPN; 2 Department of Emergency Medicine, Doshi Village Clinic, Doshi, JPN; 3 Department of Emergency and Critical Care Medicine, Division of Neurosurgical Emergency, Nippon Medical School, Tokyo, JPN

**Keywords:** ards, high-oxygen environment, legionella pneumophila, pathogenicity, vv ecmo

## Abstract

*Legionella pneumophila* can cause acute respiratory distress syndrome (ARDS), which often requires intense ventilatory management. *L. pneumophila* is an aerobic bacterium that prefers a high-oxygen environment. However, existing treatment strategies, including veno-venous extracorporeal membrane oxygenation (V-V ECMO), have not considered the pathogen’s preference for high-oxygen environments. Herein, we report two cases of patients with severe legionella pneumonia treated with V-V ECMO. The treatment strategy in these two cases was to maintain the lowest possible oxygen concentrations to inhibit the activity of *L. pneumophila*. This strategy was successful, indicating that V-V ECMO may be useful to improve outcomes in severe ARDS caused by *L. pneumophila*, especially when introduced earlier.

## Introduction

*Legionella pneumophila* is a known cause of community-acquired pneumonia, which was reported for the first time in the 1970s [1]. *L. pneumophila* tends to cause acute respiratory distress syndrome (ARDS), with a mortality rate of approximately 10-27％ [2]. Mechanical ventilation in ARDS creates a high-oxygen environment in the lungs.

Recent reports have explored the potential use of venous-venous extracorporeal membrane oxygenation (V-V ECMO) for treating severe legionella pneumonia [3]. ECMO involves treatment through extracorporeal circulation using artificial lungs and pumps. V-V ECMO specifically addresses severe respiratory failure without accompanying circulatory failure. It is well known that high oxygen concentrations can be harmful to the lung tissues in patients with *L. pneumophila* infections. Conversely, the bacterium is a strict aerobe, and high-oxygen environments increase its pathogenicity [4]. Existing treatment strategies, including V-V ECMO, have not considered the pathogen’s preference for high-oxygen environments. In this report, we propose a novel strategy for treating legionella pneumonia using low partial pressure of oxygen in V-V ECMO, considering the bacterium’s characteristics and its propensity to flourish in high-oxygen conditions.

## Case presentation

Case 1

**Figure 1:**
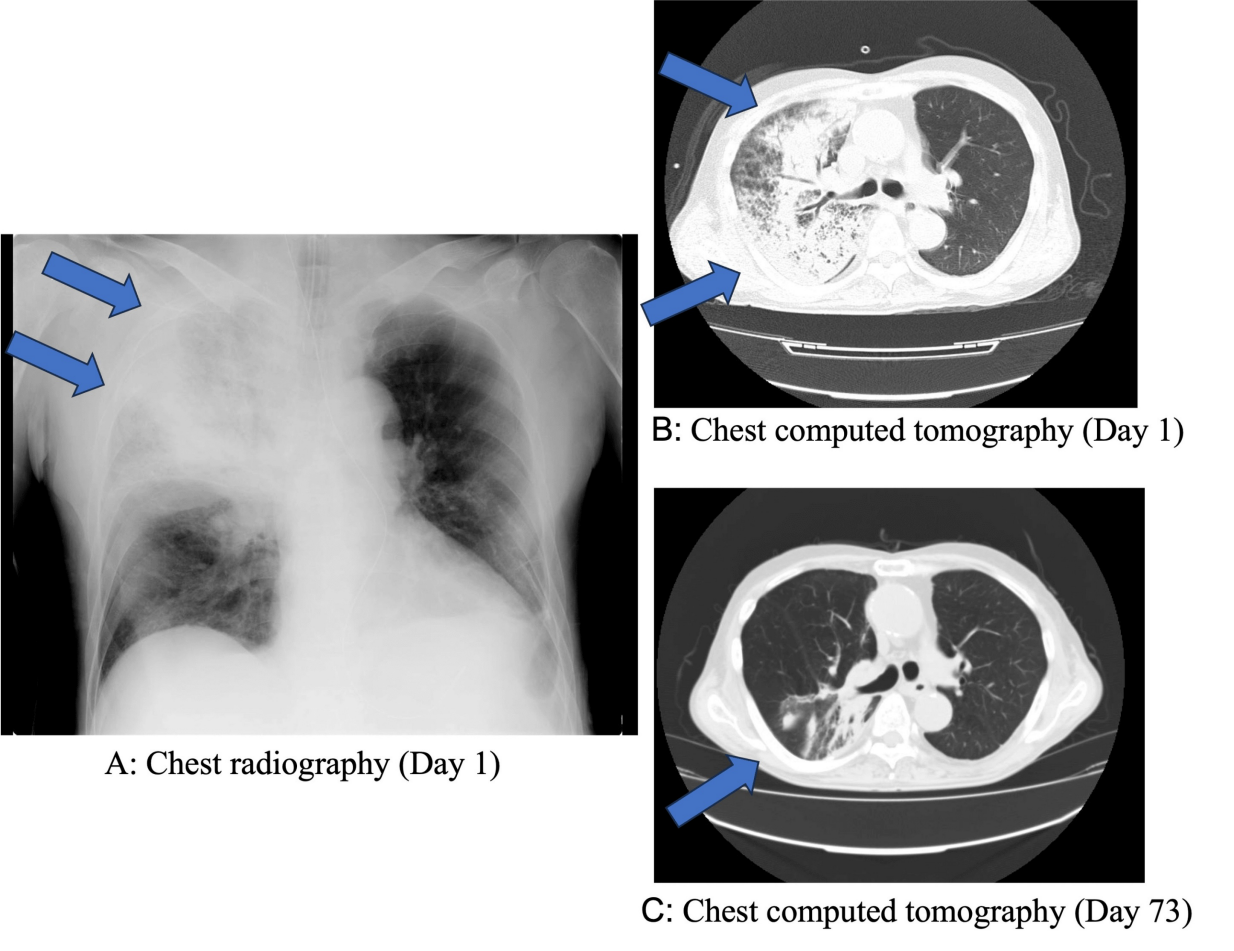
Chest radiograph and computed tomography (Case 1) Reticular shadow in the center of the right upper lung field (blue arrow).

Patient 1 was a 63-year-old man. The patient was admitted to the emergency department due to loss of consciousness while walking. Upon admission, chest radiography and computed tomography (CT) revealed severe pneumonia (Figure 1). The radiograph showed an infiltrative shadow on the right side. Antibiotics were administered from the first day of his admission to the intensive care unit. Urine examination indicated Legionella antigen positivity on the first day of admission. Because of his unstable respiratory condition, emergency intubation was performed on the first day of hospital admission. Blood tests conducted on the first day of hospitalization are detailed in Table 1.

**Table 1:** Blood test on the first day (Case 1) CPK: Creatine phosphokinase; APTT: activated partial thromboplastin time; FDP: fibrin degradation product; GOT: glutamate oxaloacetate transaminase; GPT: glutamate-pyruvate transaminase; BUN: blood urea nitrogen; CRP: C-reactive protein

Blood count	Standard value	Biochemistry	Standard value	Coagulation	Standard value
WBC 27020 /μL	3300〜8600	GOT 63 U/L	5〜37	PT (INR) 1.11 s	1±0.1
RBC 428×10^4^ /μL	435×10^4^〜555×10^4^	GPT 49 U/L	6〜43	APTT 37.6 sec	24〜34
Hb 13.9 g/dL	13.7〜16.8	CPK 658 U/L	57〜240	D-dimer 2.2 μg/mL	＜１
Ht 39.2 %	40.7〜50.1	LDH 358 U/L		FDP 10.5 μg/mL	＜５
Plt 21.5 ×10^4^ /μL	15.8 ×10^4^〜34.8 ×10^4^	AMY 46 U/L	43〜124		
		Na 125 mmol/L	135〜145		
Arterial blood gas (10L)		K 3.1 mmol/L	3.5〜5.0		
pH 7.605	7.36〜7.44	Cl 83 mmol/L	96〜107		
pCO_2_ 17.5 mmHg	35〜45	TP 6.6 g/dL	6.5〜8.5		
pO_2_ 49.3 mmHg	80〜100	Alb 2.6 g/dL	4.0〜5.2		
HCO^3-^ 17.5 mmol/L	22〜26	BUN 18.3 mg/dL	9〜21		
BE -1.9 mmol/L	−2〜＋2	Cr 1.29 mg/dL	0.6〜1.0		
Lac 60 mg/dl	4〜14	CRP 36.86 mg/dL	＜0.30		

**Figure 2:**
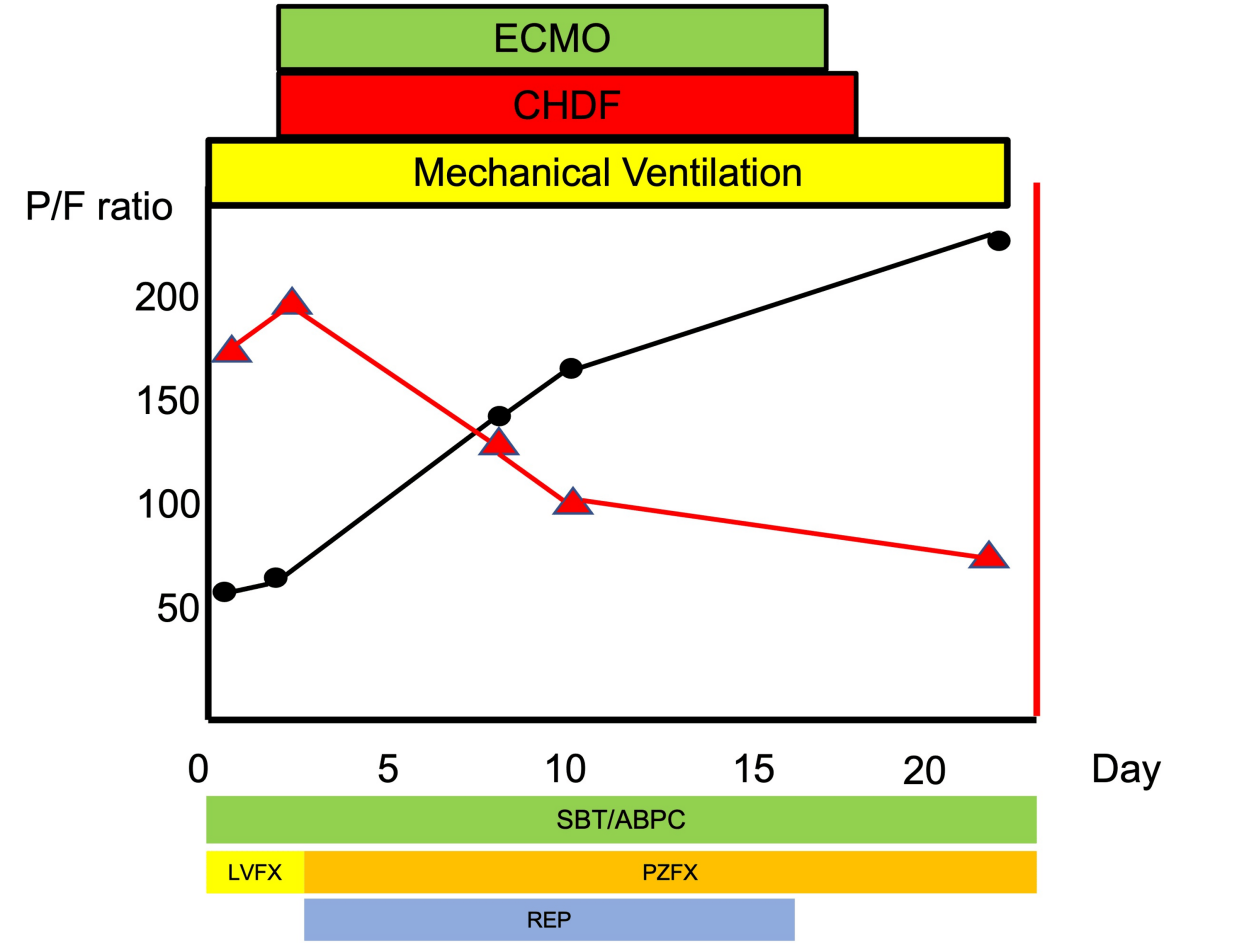
The clinical course of the patient (Case 1) ECMO: Extracorporeal membrane oxygenation; CHDF: continuous hemodialysis and filtration; SBT/ABPC: sulbactam/ampicillin; LVFX: levofloxacin; PZFX: pazufloxacin; REP: rifampicin The vertical axis and black line represent the PaO2/FiO2 ratio, whereas the horizontal axis displays the FiO2 values in the ventilator settings. The top row details respiratory, dialysis, and additional treatments; the bottom row lists antibiotics used. The ventilator’s FiO2 was successfully reduced at an early stage.

After hospitalization, his respiratory condition worsened, and the efficacy of the mechanical ventilation was limited. Therefore, V-V ECMO was initiated two days after admission. Immediately before V-V ECMO introduction, the ventilator settings were as follows: positive end-expiratory pressure (PEEP) 16 cmH2O and PaO2/FiO2 ratio 57.5. Under V-V ECMO, his respiratory condition improved. We were able to lower the FiO2 on the ventilator relatively early. After 17 days of admission, the V-V ECMO was discontinued, and after 26 days, a tracheostomy was performed. Continuous hemodiafiltration was required from day 3 to day 18, and hemodiafiltration was required from day 33 to day 41 due to acute kidney injury. Although the V-V ECMO was withdrawn, ventilator weaning took time and was achieved on day 42 because of repeated pneumonia, pleural effusions, and atelectasis. The patient was transferred to a general ward on day 49 and was discharged home on day 77 after rehabilitation. Chest CT on the 73rd day after admission revealed that the severe infiltrative shadow was smaller (Figure 1). The clinical course is shown in Figure 2. Under V-V ECMO, this patient’s respiratory condition improved.

Case 2

**Figure 3:**
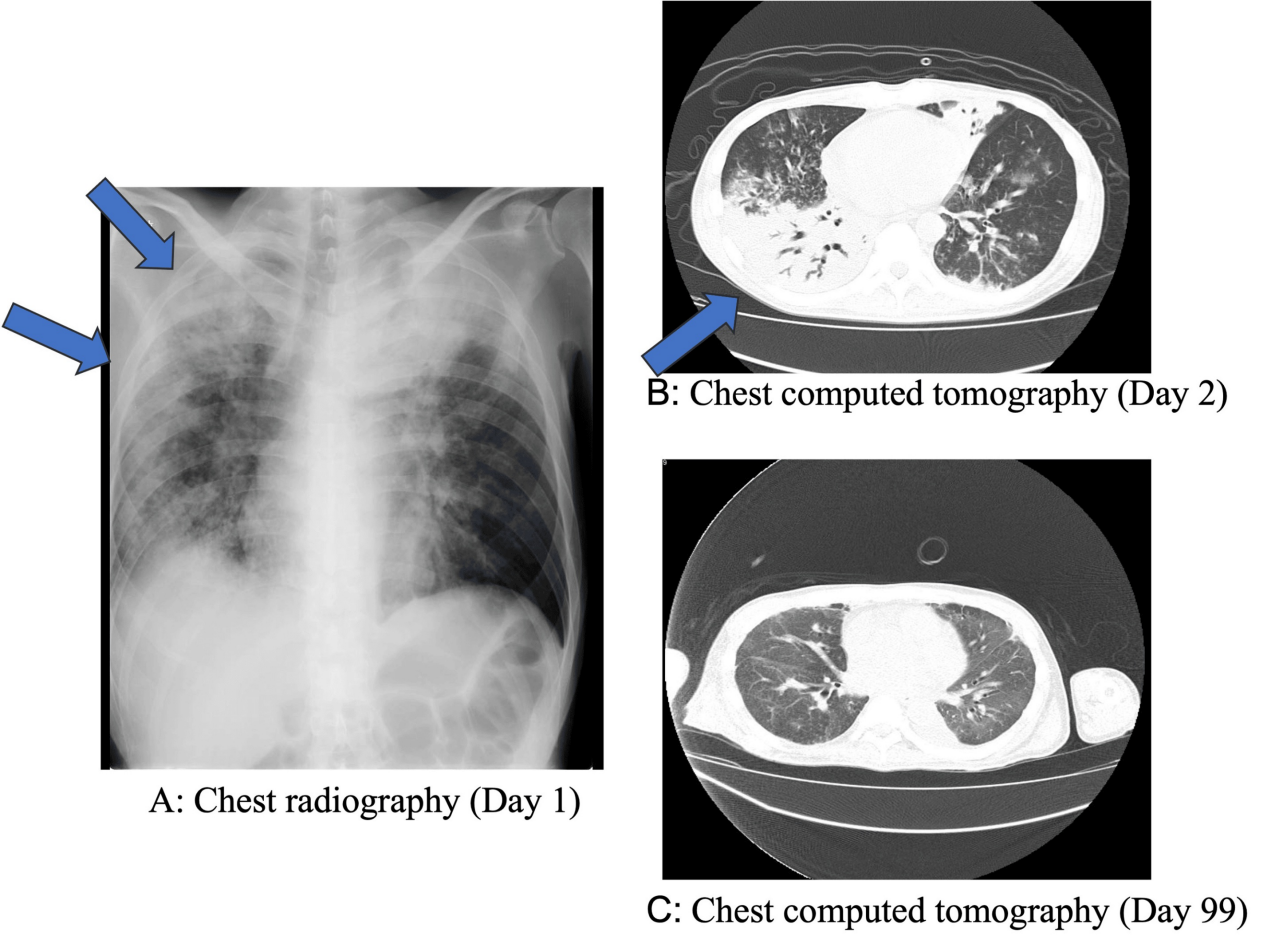
Chest radiography and computed tomography (Case 2) An infiltrative shadow (blue arrow) with bronchial translucency is present mainly in the dorsal right lung field on days 1 and 2.

**Figure 4:**
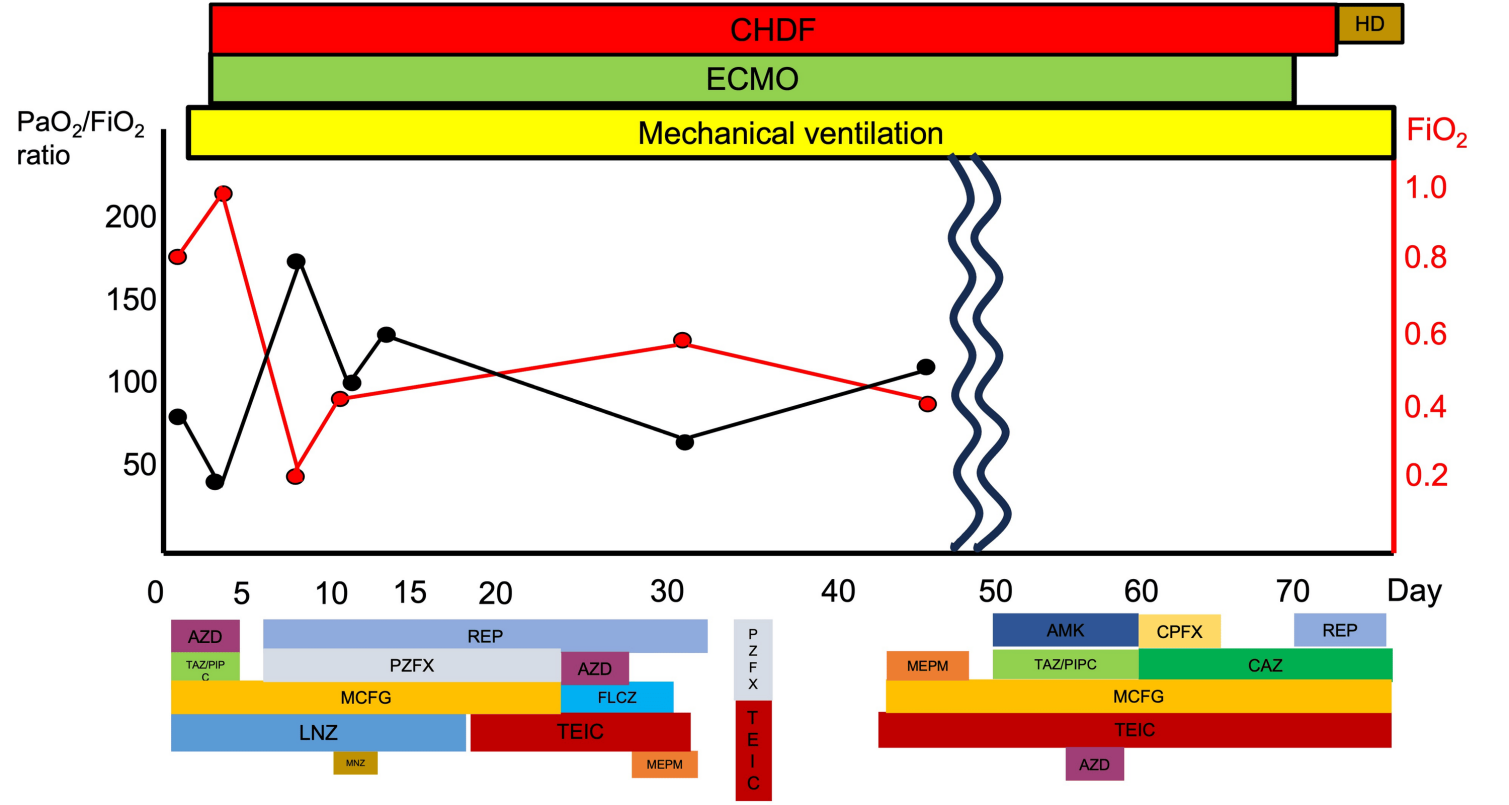
The clinical course of the patient (Case 2) ECMO: Extracorporeal membrane oxygenation; CHDF: continuous hemodialysis and filtration; HD: hemodialysis; AZM: azithromycin; TAZ/PIPC: tazobactam/piperacillin; PZFX: pazufloxacin; REP: rifampicin; MCFG: micafungin; FLCZ: fluconazole; LNZ: linezolid; TEIC: teicoplanin; MNZ: metronidazole; MEPM: meropenem; CFPX: ciprofloxacin; CAZ: ceftazidime The vertical axis and black line indicate the PaO2/FiO2 ratio, whereas the horizontal axis displays the FiO2 values in the ventilator settings. The top row details respiratory, dialysis, and other treatments; the bottom row lists the antibiotics used. Although the ventilator’s FiO2 can be lowered relatively early in these patients, the duration of ventilator weaning and rehabilitation tends to be long.

The patient was a 47-year-old man who reported a loss of appetite for a month and experienced dyspnea three days before emergency transportation and presentation to the hospital. Upon admission, he received oxygen therapy and antibiotics. Blood tests conducted on the first day of hospitalization are detailed in Table 2. On the first and second days of hospital admission, chest radiography and CT revealed severe pneumonia (Figure 3). Because of his worsening respiratory condition, tracheal intubation was performed. After five days of admission, V-V ECMO was initiated to limit the use of mechanical ventilation. Immediately before the V-V ECMO was introduced, the ventilator settings were as follows: PEEP 15 cmH2O and PaO2/FiO2 ratio 34. The antibody titer for Legionella was high, confirming a diagnosis of legionella pneumonia. After two V-V ECMO exchanges, the patient’s general condition and respiratory function improved. On day 70 of admission, the V-V ECMO was discontinued. The patient had severe post-inflammatory interstitial changes; there was development of multi-drug-resistant Pseudomonas aeruginosa infection and difficulty in regulating the body's water balance due to renal dysfunction made ECMO weaning a slow process. The chest CT performed on the 99th day after admission is shown in Figure 3. Following long-term rehabilitation, the patient was discharged 175 days after admission. The clinical course is shown in Figure 4. The figure shows a lower fraction of inspired oxygen (FiO2) after the introduction of V-V ECMO.

**Table 2:** Blood test on the first day (Case 2) CPK: Creatine phosphokinase; APTT: activated partial thromboplastin time; FDP: fibrin degradation product; GOT: glutamate oxaloacetate transaminase; GPT: glutamate-pyruvate transaminase; BUN: blood urea nitrogen; CRP: C-reactive protein

Blood count	Standard value	Biochemistry	Standard value	Coagulation	Standard value
WBC 14890 /μL	3300〜8600	GOT 21 U/L	5〜37	PT (INR) 1.68 sec	1±0.1
RBC 314×10^4^ /μL	435×10^4^〜555×10^4^	GPT 26 U/L	6〜43	APTT 34.7	24〜34
Hb 8.4 g/dL	13.7〜16.8	CPK 21 U/L	57〜240	D-dimer 211.0 μg/mL	＜１
Ht 28.0 %	40.7〜50.1	LDH 199U/L		FDP 26.6 μg/mL	＜５
Plt 66.1×10^4^ /μL	15.8×10^4^〜34.8 ×10^4^	AMY 49 U/L	43〜124		
		Na 137mmol/L	135〜145		
Arterial blood gas (10L)		K 5.0mmol/L	3.5〜5.0		
pH 7.480	7.36〜7.44	Cl 101 mmol/L	96〜107		
pCO_2_ 33.1 mmHg	35〜45	TP 7.0g/dL	6.5〜8.5		
pO_2_ 68.0mmHg	80〜100	Alb 1.7 g/dL	4.0〜5.2		
HCO^3-^ 24.3mmol/L	22〜26	BUN 19.5 mg/dL	9〜21		
BE 1.3mmol/L	−2〜＋2	Cr 0.74 mg/dL	0.6〜1.0		
Lac 12 mg/dl	4〜14	CRP 40.89 mg/dL	＜0.30		

## Discussion

In this report, we present two cases of patients with severe legionella pneumonia who were treated with V-V ECMO to maintain low oxygen concentrations to inhibit the activity of L. pneumophila.

L. pneumophila is a gram-negative bacterial pathogen known to cause severe life-threatening lobar or atypical pneumonia. It can be contracted during traveling, particularly to hot springs, where L. pneumophila is detected in about 40% of the water. Antibiotics, particularly azithromycin (+ rifampicin), are mainstays for legionella pneumonia treatment. When a patient has acute lung injury from L. pneumophila infection, mechanical ventilation is often required.

V-V ECMO is typically useful for treating severe pneumonia and respiratory failure, such as in influenza virus or SARS-CoV-2 infections [3,4]. The purpose of the V-V ECMO is to manage hypoxemia and allow the lungs to rest. While there are case reports documenting successful legionella pneumonia treatment using V-V ECMO, some reports [3] indicate that 75% of the patients treated using V-V ECMO for *L. pneumophila*-associated ARDS were weaned from the V-V ECMO, and 67% survived until hospital discharge.

L. pneumophila is a strict aerobe, preferring higher oxygen concentrations. While a previous case report suggested that V-V ECMO is useful for treating severe L. pneumophila infections [5], no strategy for this treatment had been proposed. A study reported that hyperoxia was an important factor affecting acute lung injury in L. pneumophila-infected mice. Through death receptor-mediated signals, apoptosis increases the risk of acute lung injury [6]. Another study showed that Fas-mediated signaling apoptosis increased acute lung injury caused by L. pneumophila in hyperoxic environments [7].

Although it has been shown in animal experiments that high-oxygen concentration environments can lead to worsening conditions for patients with L. pneumophila infections, the effects on living organisms have not been demonstrated. However, considering the fact that the causative bacteria are aerobic, V-V ECMO may be useful to avoid high-oxygen concentration inhalation when necessary. In the present report, low-oxygen concentration V-V ECMO was administered on the third day in Case 1 and on the second day in Case 2. The strategy behind using V-V ECMO is to provide “lung rest,” preventing barotrauma caused by high PEEP during mechanical ventilation. The standard protocol involves maintaining a low tidal volume. In addition to this basic strategy, the FiO2 must be lowered gradually to decrease the pathogenesis of L. pneumophila.

Despite the risks associated with V-V ECMO, primarily from 16-22 Fr catheter insertion, its administration in the early stages of infections has proven to be effective. Therefore, the administration of V-V ECMO within the first few days of hospitalization should be considered for patients requiring mechanical ventilation in this early period. 

In the presented cases, a low oxygen concentration was maintained in the lungs. While the environment around L. pneumophila may reduce the efficacy of this strategy, this report suggests that the best management strategy for severe L. pneumophila infection is V-V ECMO, along with strict antibacterial treatment. The introduction of V-V ECMO is useful for reversible lung diseases, such as bacterial and viral diseases. In the case of L. pneumophila infection, V-V ECMO should be introduced before increasing the oxygen delivery via the ventilator. Although the introduction of V-V ECMO was originally based on the Extracorporeal Life Support Organization guidelines (e.g., ventilator settings) and is often done after some determination of antibiotic efficacy, we believe that an earlier introduction is necessary considering the virulence of L. pneumophila.

## Conclusions

We present two cases of severe *L. pneumophila *infection that were treated using V-V ECMO to maintain a low FiO2. It is important to understand the characteristics of *L. pneumophila* infection, which is aerobic, and considering early ECMO introduction to reduce the inhaled oxygen concentrations might be useful. Further case accumulation is needed.
